# Discrete, recurrent, and scalable patterns in non-operant judgement underlie affective picture ratings

**DOI:** 10.1007/s10339-024-01250-9

**Published:** 2024-12-07

**Authors:** Leandros Stefanopoulos, Byoung-Woo Kim, John Sheppard, Emanuel A. Azcona, Nicole L. Vike, Sumra Bari, Shamal Lalvani, Sean Woodward, Nicos Maglaveras, Martin Block, Aggelos K. Katsaggelos, Hans C. Breiter

**Affiliations:** 1 Department of Electrical and Computer Engineering, Northwestern University, Evanston, IL USA; 2 School of Medicine, Aristotle University of Thessaloniki, Thessaloniki, Greece; 3 Departments of Computer Science and Biomedical Engineering, University of Cincinnati, Cincinnati, OH USA; 4 Department of Internal Medicine, Yale School of Medicine, New Haven, CT USA; 5 Feinberg School of Medicine, Northwestern University, Evanston, IL USA; 6 Integrated Marketing Communications, Northwestern University, Evanston, IL USA; 7 Department of Computer Science, Northwestern University, Evanston, IL USA; 8 Department of Radiology, Feinberg School of Medicine, Northwestern University, Evanston, IL USA; 9 Laboratory of Neuroimaging and Genetics, Department of Psychiatry, Massachusetts General Hospital and Harvard School of Medicine, Boston, MA USA

**Keywords:** Relative preference theory, Liking, Reward, Aversion, Preference, Approach, Avoidance, Big data, Judgment

## Abstract

Operant keypress tasks in a reinforcement-reward framework where behavior is shaped by its consequence, show lawful relationships in human preference behavior (i.e., approach/avoidance) and have been analogized to “wanting”. However, they take 20–40 min as opposed to short non-operant rating tasks, which can be as short as 3 min and unsupervised, thus more readily applied to internet research. It is unknown if non-operant rating tasks where each action does not have a consequence, analogous to “liking”, show similar lawful relationships. We studied non-operant, picture-rating data from three independent population cohorts (*N* = 501, 506, and 4019 participants) using the same **7**-point Likert scale for negative to positive preferences, and the same categories of images from the International Affective Picture System. Non-operant picture ratings were used to compute location, dispersion, and pattern (entropy) variables, that in turn produced similar value, limit, and trade-off functions to those reported for operant keypress tasks, all with individual *R*^2^ > 0*.*80. For all three datasets, the individual functions were discrete in mathematical formulation. They were also recurrent or consistent across the cohorts and scaled between individual and group curves. Behavioral features such as risk aversion and other interpretable features of the graphs were also consistent across cohorts. Together, these observations argue for lawfulness in the modeling of the ratings. This picture rating task demonstrates a simple, quick, and low-cost framework for quantitatively assessing human preference without forced choice decisions, games of chance, or operant keypressing. This framework can be easily deployed on any digital device worldwide.

**Supplementary Information:**

The online version contains supplementary material available at 10.1007/s10339-024-01250-9.

## Introduction

**Fig. 1:**
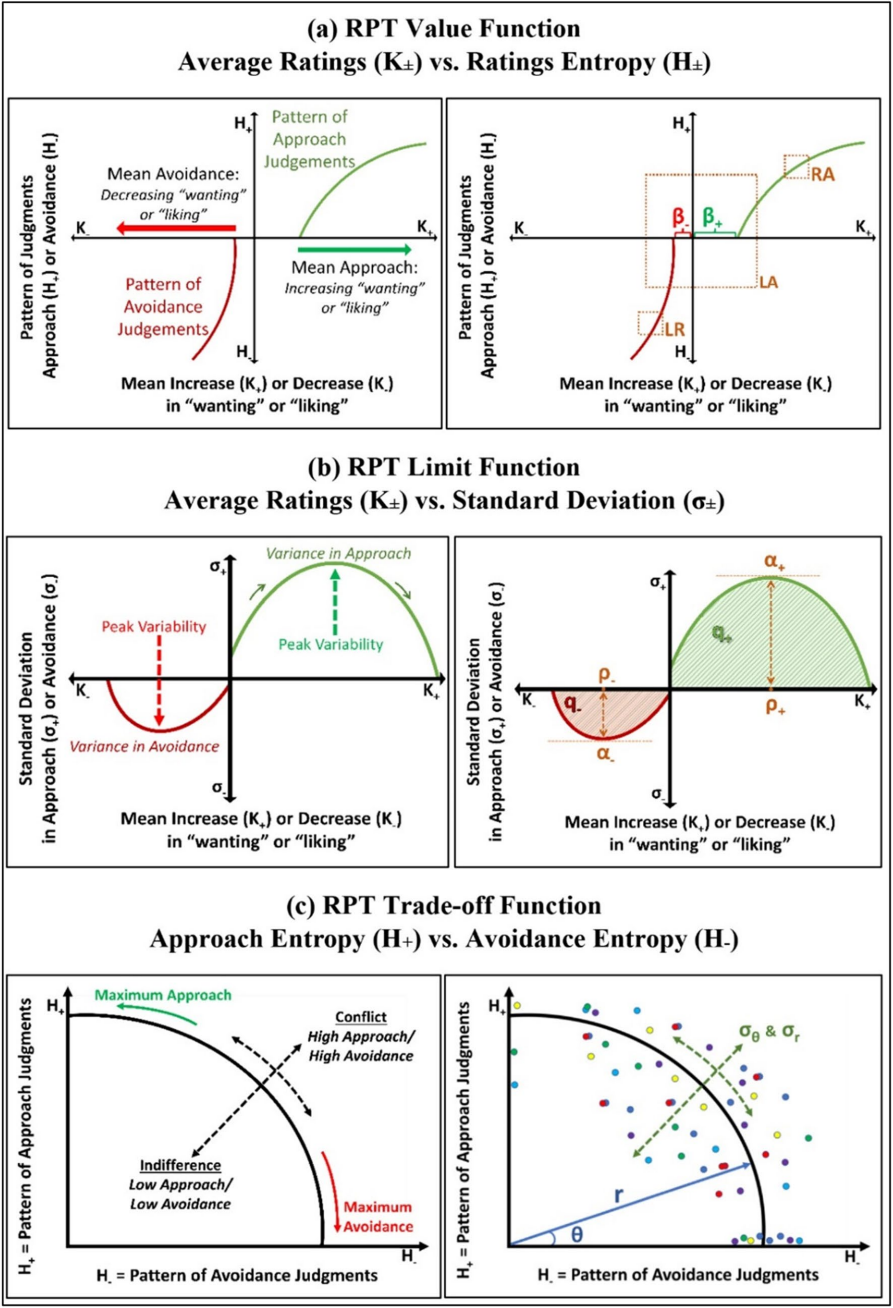
RPT is characterized in part by features that describe relationships between these three behavioral variables: {K, H, σ}. These relationships include: (a) a value function plotting the Shannon entropy (*H*_±_), against the average ratings (*K*_±_) for approach or avoidance toward a suite of objects. This function is referred to as a value function given it calibrates “wanting” or “liking” (depending on the task structure) against the pattern of previous judgements and is consistent with the prospect theory value function. Standard features of these curves, shown in the diagram, include loss aversion (*LA*) and risk aversion (*RA*) from the literature on behavioral economics. The corollary of *RA* is also shown, herein referred to as loss resilience (*LR*). Two offsets are also noted that are clear in the individual data, relating to an “approach offset” (*β*_+_) and “avoidance offset” (*β*_*−*_). (b) A variance-mean relationship is observed between the average ratings (*K*_±_) plotted against the corresponding standard deviation of rating responses (*σ*_±_). This relationship is characterized by increasing variance up to a peak followed by decreasing variance back to baseline. This function describes limits to preference or its “saturation”. Standard features of this curve include the apices of the quadratic fits, the “turning points” (*ρ*_±_) or value of *K*_±_ at which *σ*_±_ is maximal/minimal, and the quadratic areas (*q*_±_) of these curves bounded by the *K*-axis. (c) A trade-off function between the approach entropy (*H*_+_) and avoidance entropy (*H*_*−*_) was also identified, defining how bundles of approach judgments were balanced with bundles of avoidance judgments as a quantifiable trade-off between approach and avoidance. This trade-off function can be characterized by the mean polar angle of the trade-off curves (*θ*), the standard deviation of this polar angle (its dispersion) (*σ*_*θ*_), the mean radial distance for the trade-off curves (*r*), and its corresponding standard deviation (the dispersion in *r*) (*σ*_*r*_)

Preference can be defined from a psychological and neuroscience perspective as the variable extent an organism approaches or avoids events in the world, based on the rewarding or aversive effects of these events (Lewin 1935; Schneirla 1959, 1965). Preference-based behavioral variables that measure the intensity and patterns of approach/avoidance behavior with an operant keypress task based on reinforcement reward theory (Baum 1974; Herrnstein 1964) show lawful relationships in humans when using visual (Kim et al. 2010; Lee et al. 2015) and auditory stimuli (Livengood et al. 2017) (see Fig. 1). Feynman defined lawfulness by constructs such as (i) recurrency of experimental results across unrelated studies, (ii) discreteness of the mathematical formulation/description of results, and (iii) scaling of the results across two or more levels of organization (such as individual behavior and descriptions of group behavior) (Feynman 2017). These lawful behavioral relationships have been associated with activation in brain reward circuitry by use of model-based functional MRI (Aharon et al. 2001; Viswanathan et al. 2015), imaging genetics (Gasic et al. 2009; Perlis et al. 2008), and quantitative morphometry (Makris et al. 2008).

The keypress task used in such studies was derived from an operant framework (Baum 1974; Herrnstein 1964) where each keypress had an incremental consequence on stimulus view time(Aharon et al. 2001; Lee et al. 2015); this has been validated across multiple studies (Aharon et al. 2001; Elman et al. 2005; Gasic et al. 2009; Kim et al. 2010; Lee et al. 2015; Levy et al. 2008; Makris et al. 2008; Perlis et al. 2008; Strauss et al. 2005; Viswanathan et al. 2015, 2017; Yamamoto et al. 2009). The keypress task can be analogized to the construct of “wanting” as opposed to “liking” (Aharon et al. 2001; Berridge and Robinson 1995). Per foundational papers (Berridge 2006; Berridge and Robinson 1995; Robinson and Berridge 2001), “Liking” refers in general to the pleasure or displeasure experienced from consuming a reward or avoiding a sanction. It reflects the hedonic impact, the feeling of pleasure after the reward is received or aversion relating to a sanction. “Liking” is commonly juxtaposed to “wanting” which refers to the motivational drive or desire to obtain a potential reward and can be framed by the pursuit of something rewarding or avoidance of something aversive (Chillà et al. 2019; Pool et al. 2015, 2016; Wu et al. 2018) as by an operant keypress paradigm (Livengood et al. 2017; Viswanathan et al. 2017).

In humans, reinforcement reward tasks with operant keypressing can be framed by variables that quantify the average (mean) magnitude (*K*), variance (*σ*), and the pattern (i.e., Shannon entropy (*H*)) of participants’ keypress-based behavior. We refer to this methodology, and the multiple relationships between these variables and features based on their graphical relationships, as relative preference theory (RPT) (see Fig. 1). Two of the graphs produced for RPT resemble graphs derived with different variables from prospect theory (Kahneman and Tversky 1979) and the mean–variance function described by Markowitz for portfolio theory (Markowitz 1952). Given this graphical resemblance, it is important to note that the definition of preference in psychology and economics carries different meanings. In psychology, preferences associated with ‘wanting’ or ‘liking’ are framed by judgments that precede decisions, which can be quantified by reinforcement reward or incentive reward tasks (Baum 1974; Berridge and Robinson 1995; Breiter et al. 2015; Dai et al. 2010; Herrnstein 1964; Kim et al. 2010; Montague 2008). In economics, preferences are relations derived from consumer choice data (see the axioms of revealed preference (Mas-Colell et al. 1995)) and reflect decision-making. The economist Paul Samuelson noted that ‘Utility is assumed to be correlative to Desire or Want,’ (Marshall 1890) and in this paper, we deal with judgments rather than choices, which we presume to be related (Breiter et al. 2015; Montague 2008). Instead of collecting choice data, we follow previous approaches of extracting value variables with rating data, such as implemented by Kahneman (Breiter et al. 2001). In this regard, we emphasize that our functions quantifying value are not to be interpreted as the standard representations of a preference relation in economics (and hence, extracted parameters such as loss aversion and risk aversion are not to be interpreted in the same context); going forward, we will refer to judgment-based loss aversion as LA, and judgment-based risk aversion as RA.

To date, RPT has only been discussed in an operant framework where effort traded for viewing time can be considered a model of “wanting” (Aharon et al. 2001), as opposed to a stimulus–response or non-operant framework where individuals see a stimulus and make a response for which there are no consequences (i.e., a change in viewing time). In a recent study, RPT in a keypress framework was compared to a prospect theory framework in which ratings were made under conditions of uncertainty during a game of chance (i.e., anticipation phase of the trial with a spinner), and under conditions of certainty when the outcome was revealed (i.e., outcome phase of the trial when the spinner landed on a specific outcome). During anticipation, ratings produced statistically similar LA measures to those of keypressing with an RPT analysis, whereas during the outcome phase, ratings showed no overweighting of losses relative to gains (Lee et al. 2015). *LA* was specifically defined by Tversky and Kahneman (1992) to describe an overweighting of negative judgements relative to positive ones under conditions of risk. These observations (Lee et al. 2015) raised the hypothesis that in a non-operant model where actions have no consequences (i.e., “liking”), a rating task with no uncertainty might produce RPT-like curves, but not show the same degree of overweighting of losses relative to gains which characterize *LA* during uncertainty. Rating tasks (non-operant) can be completed in 3–5 min as opposed to keypressing tasks (operant) which can take 20–40 min, and rating tasks can be fully unsupervised. Rating tasks have already been shown to produce strong data for machine learning of medical judgment such as vaccine choice (Vike et al. 2024) and mental illness (Bari et al. 2024; Lalvani et al. 2024; Stefanopoulos et al. 2024). Demonstrating that rating tasks show consistent law-like patterns (Feynman 2017) is important for framing their broader use for machine learning applications to mental illness and medical treatment. These considerations led us to analyze three separate cohorts of human participants using a rating task and picture stimuli from the International Affective Picture Set (IAPS) (Lang et al. 1997, 2008). To quantify the similarity of potential rating-based curves across distinct experiments, we framed 15 metrics that included *LA*, *RA*, and the equivalent of *RA* on the avoidance value function (see Fig. 1a). Twelve other metrics were defined from standard curve features such as offsets (see Fig. 1a), apices, *x*-axis values for the apices, and quadratic areas (see Fig. 1b), along with mean and variance measures for any angles or radial distances (see Fig. 1c) (see Methods). These 15 extracted “features” were not considered definitive for reconstituting each function but could be psychologically interpreted (see Methods).

Three hypotheses were raised: (1) Would ratings, in the absence of the operant framework, produce preference relationships similarly to what has been observed with keypressing? (see Fig. 1) (2) If ratings produced RPT-like curves, would these functions be (i) mathematically discrete, (ii) recurrent across cohorts, and (iii) scale from individual to group data? Namely, would functions from ratings meet three of the primary criteria raised by Feynman for lawfulness? (Feynman 2017) (3) How consistent would rating-based curves be to each other if they came from distinct cohorts and experimental sessions? Would features of these functions, resembling constructs such as LA and RA from behavioral economics and Prospect Theory, or resembling Markowitz’s decision utility around variance-mean functions (Markowitz 1952) be similar between distinct cohorts? Furthermore, would the extracted behavioral features from these functions potentially differ from previously published ones computed from keypressing tasks? From this work, we found that judgment features extracted from rating task curves resembled published curves from operant keypressing (e.g., Kim et al. 2010), and met three criteria for lawfulness per Feynman (2017). These judgment features were consistent across three distinct experiments, and thus provide a platform for large-scale study of population samples for machine learning applications, especially those in the domain of mental health and allowing interpretation of human behavior in general (Lalvani et al. 2024; Stefanopoulos et al. 2024; Vike et al. 2024).

## Methods

### Participants

In all three studies, rating and survey responses were collected online to meet demographic criteria established by the United States (U.S.) census. One study involved 501 participants for the Emotion and Behavior Study (EBS), an online study of U.S. adult (i.e., ≥ 18 years of age) consumers, conducted by Research Results, Inc. (Boston, MA) from July 9 to August 9, 2016. The second study consisted of 506 participants randomly sampled from the general U.S. population using a participant database accessed by Gold Research Inc. (San Antonio, Texas) for the Automated Mental Health Assessment Study (AMHA), referred to as the AMHA-1 cohort. Questionnaire responses for AMHA-1 were collected in 2021 between February 15 and March 01, approximately one year following the official COVID-19 pandemic declaration in the U.S. The third study involved 4,019 participants, also randomly sampled from the general U.S. population using a participant database accessed by Gold Research Inc. for the AMHA study, referred to as the AMHA-2 cohort, the study took place from December 01 to December 30, 2021. Please see references (Bari et al. 2022; Woodward et al. 2022) for details about recruitment and consenting procedures. All participants signed a written consent form for their response data to be used, and data released to Northwestern University from Gold Research Inc. and Research Results Inc. were anonymized.

Participant demographic information includes gender identity, age group (in years), employment status, education level, handedness, and race/ethnicity as summarized in Table 5 (Appendix), including percent compositions for each group within each corresponding demographic measure.

### Picture stimuli

Stimulus sets across the rating task consisted of images from the IAPS (Lang et al. 1997, 2008), a well-validated emotional stimulus set. For all cohorts, six categories of pictures were used: (1) sports, (2) disasters, (3) cute animals, (4) aggressive animals, (5) nature (beach vs. mountains), and (6) people in bathing suits, with eight pictures per category (48 pictures in total). These picture categories were selected to generally cover the valence-arousal space; they thus invoked a range of high/low valence (positive/negative) and high/low arousal (intensity of activation) feelings. This approach was used to allow a broad framework for assessing judgment, as has been successfully applied for the prediction of vaccine uptake and three mental health conditions (Bari et al. 2024; Lalvani et al. 2024; Stefanopoulos et al. 2024; Vike et al. 2024). All stimuli used had mild to moderate levels of arousal per initial validation assessments (Lang et al. 1997, 2008), and each picture category framed a small range of negative or positive arousal (please see Kim et al. 2010). Pictures had a maximum size of 1*,*204 × 768 pixels in all three studies. All picture stimulus sets reported in the present study are collectively referred to as “IAPS stimuli” throughout the text.

### Picture rating task (“liking” assessment)

Participants were prompted for the rating task while completing an online digital survey, which contained questionnaires regarding participant demographic information and research questionnaires for depression symptoms using the Patient Health Questionnaire (PHQ-9) (Kroenke et al. 2001); trait anxiety using the Spielberger State-Trait Anxiety Inventory (STAI) (Spielberger et al. 2001); a broad array of mental health, neurological, and medical issues using the MGH Phenotype Genotype Project in Addiction and Mood Disorders symptom questionnaire (MGH-SQ); and behavioral health disorders (e.g., internalizing or externalizing psychiatric disorders, substance use disorders, or crime/violence problems) from the GAIN-SS short screen assessment(Dennis et al. 2006). For the picture rating task, the instructions presented to participants for each study followed a standard text, referenced in: Vike et al. 2024; Lalvani et al. 2024; and Bari et al. 2024, and based on:

*The next part of this survey involves looking at pictures and then responding how much you like or dislike the image. Please rate each image on a scale from -3 (Dislike Very Much) to + 3 (Like Very Much). Zero (0) is neutral... meaning you have no feelings either way. The images are a set of photographs that have been used by scientists around the world for over 20 years.*

*It is important you rate each picture based on your initial emotional response.*

*There are no right or wrong answers... just respond with your feelings and rate the pictures very quickly. Please click “Next” to begin.*

Each picture was presented as shown in Fig. 5, where the ratings below each picture were selectable using the mouse cursor or keyboard arrows. There was no time limit for assigning ratings to each picture, but participants were requested to rate each picture as quickly as possible, and they were not able to change their response after selecting a rating. After each rating selection was made, the next picture was automatically loaded and presented. This online platform was provided by Research Results (EBS) and Gold Research (AMHA1 and AMHA2) and was accessible across digital devices, including laptops, tablets, and smartphones, ensuring a responsive design for easy use on any screen size. Figure 5 provides an example of a typical rating prompt, where an image is displayed along with the available rating options.

### Data quality screening

Data integrity was assessed for all data from the three studies. Quality assurance was conducted based on four exclusion criteria for picture rating tasks and survey data (survey-based non-rating data is not described herein), which reduced the analysis to 281 participants for the EBS cohort, 366 for AMHA-1, and 3476 for AMHA-2. These four exclusion criteria were:

1. Participants selected the same response throughout any section of the questions/tasks (e.g., selecting option “1” for all questions),
2. Participants indicated they had ten or more clinician-diagnosed illnesses (data not described here),
3. Participants showed minimal variance in a picture rating task (i.e., all pictures were rated the same or varied only by one point), and
4. If *both* education level and years of education did not match *and* if they completed the questionnaire in less than 500 s (800 s for the AMHA-2 cohort).

Further quality assurance involved assessment of RPT variables and curves from the picture rating tasks. Variables that were quantified included the average magnitude of ratings (*K*), variance in ratings (*σ*), and the pattern of ratings or information (i.e., Shannon entropy (*H*)). *K* reflected the average (mean) of positive ratings a subject made (*K*_+_) or negative ratings (*K*_*−*_) within each picture category. Other metrics included the variance in positive ratings (*σ*_+_) or negative ratings (*σ*_*−*_), along with the Shannon entropy [i.e., information (Shannon and Weaver 1949)] of positive ratings (*H*_+_) or negative ratings (*H*_*−*_) for stimuli within each category. The Shannon entropy is a core variable in information theory that characterizes the degree of uncertainty across a set of responses (Shannon and Weaver 1949); it quantifies the pattern of judgements made to a set of stimuli and could thus be considered a memory variable. Collectively, these variables capture judgments about the valence of judgement (positive vs. negative or approach vs. avoidance) as well as its magnitude (intensity of rating) to describe relative preferences (Breiter and Kim 2008; Kim et al. 2010) (See Fig. 1).

When evaluating data quality, raw data was assessed for cases when K = 0 for a given category (i.e., cases where the subject made all neutral ratings to neither approach nor to avoid any stimulus in the category). Computing the Shannon entropy, H, for a given picture category requires that K > 0 given that when K = 0, the H computation results in evaluating log ($\frac{0}{0}$) which is undefined. In these cases, the Shannon entropy was set to H = 0 for categories in which the subject rated “0” for all the stimuli.

Before carrying out the RPT analyses, and fitting models to participants’ ratings, data was further screened for additional criteria beyond when *K* = 0 for a given category. The complete set of model fit inclusion/exclusion criteria was as follows:

1. Valid entropy (H) calculations (see prior paragraph),
2. Exclusion of data points lying beyond three times the interquartile range (IQR), below the first quartile or above the third quartile (i.e., removing extreme outliers),
3. Sufficient number of data points to fit the model with a computable ${R}^{2}$ (e.g., at least three points for a non-linear fit), and
4. Coherence of model fits between individual and group data. This last criterion required that the curve direction for individual subject fits be consistent with the curve direction of the group-level statistical fits (and boundary envelopes), and therefore corroborate most of the observed subject data.

Criteria (3) and (4) are necessary operational definitions for quality assurance given the potential for convergence failures with curve fitting. For the AMHA- 2 cohort, criteria (2) was not implemented given the potential for greater variance than with the prior two studies due to the COVID-19 pandemic; in lieu of a 3 × IQR threshold, a threshold was set for the two curve features with a small number of very extreme outliers: loss aversion (*LA* > 200, resulting in *N* = 42 exclusions) and positive quadratic area (*q*_+_  > 100, resulting in *N* = 5 exclusions) (see definitions in Relative preference analysis below).

In total, multiple types of model fitting was performed for the rating data, following extensive experimentation with a range of potential models (Kim et al. 2010): group and individual models for the (*K,* *H*) data, (*K,* *σ*) data, and (*H*_*−*_*,* *H*_+_) data distributions. We used the models with the highest R^2^ and simplest formulation for structure observed between K, H, and *σ* variables. Each of these fits allows distinct types of nonlinear relationship in the data to be captured that complement each other regarding the calibration of value, limits to value, and tradeoffs between positive and negative values (Kim et al 2010). For the group data, we generated group-level data fits along with boundary envelopes (power-law fits and logarithmic fits for group (*K,* *H*) data), and quadratic fits for group (*K,* *σ*) data to guide the focus of statistical testing based on the power-law fits (*K,* *H*), and quadratic fits (*K,* *σ*) for individual data. Individual data then followed these fits based on logarithmic and simple power-law fits for individual (*K,* *H*) value functions, quadratic fits for individual (*K,* *σ*) limit functions, and radial fits for individual (*H*_*−*_*,* *H*_+_) trade-off distributions(Kim et al. 2010; Livengood et al. 2017).

### Relative preference analysis

Initial analysis of picture rating data involved qualitative assessment of the mean positive and negative ratings for each category of picture to confirm there were no major deviations in IAPS stimuli ratings across the three studies.

For the relative preference analysis, we replicated the methodology described in detail in references (Kim et al. 2010), (Livengood et al. 2017), (Viswanathan et al. 2017). We used the iterative modeling approach of reference (Banks and Tran 2009) to identify RPT patterns in the data and three of the four putative signatures of lawfulness, as described previously with visual (Breiter and Kim 2008; Kim et al. 2010) and auditory stimuli (Livengood et al. 2017). We thus sought “discrete” mathematical fitting of patterns within the data, “recurrence” of patterns across the three distinct experimental cohorts, and “scalability” of the observed patterns. We utilized datasets that met stringent criteria for quality assurance, then assessed the graphical structure between the three behavioral variables, {K, H, σ}. For the rating tasks, these variables reflected the mean positive ratings or negative ratings within a picture category (*K*_±_), the Shannon entropy of positive/negative ratings within a category (*H*_±_), and the standard deviation (*σ*_±_) of positive/negative ratings within a category. Graphical analyses sought to determine the presence of functions, manifolds, or boundary envelopes to individual, and separately, group data that were graphically similar to RPT functions, manifolds, and boundary envelopes (Breiter and Kim 2008; Kim et al. 2010; Lee et al. 2015; Livengood et al. 2017).

Formal testing of discreteness, recurrence, and scaling was done following procedures in other published papers (Bari et al. 2024; Kim et al. 2010; Lalvani et al. 2024; Lee et al. 2015; Livengood et al. 2017; Stefanopoulos et al. 2024; Vike et al. 2024; Viswanathan et al. 2015, 2017). To assess if mathematical fitting was discrete, the goodness of fit for the (*K,* *H*) value functions and (*K,* *σ*) limit functions, across the three experiments, were characterized by *R*^2^, and adjusted *R*^2^ statistics; then tabulated by location and dispersion estimates. Given prior keypress findings of discreteness with *R*^2^ > 0*.*7, we assessed if definable functions for individual data and manifold fits (and/or boundary envelopes) for group data had clear parameter estimates and showed *R*^2^ > 0*.*7. For recurrence, we assessed if similar individual and group models were observed for each of the three independent populations, and if the extracted RPT features (*N* = 15) for individual functions were similar across the three groups. Lastly, scale invariance and simple power-law fitting was assessed by performing linear regressions following logarithmic transformations of both the *K*- and *H*-axes. If the resulting fits characteristically demonstrated asymptotic behavior (0 < *a* < 1, given *H*(*K*) = *bK*^*a*^), this implied that substantial changes in the input variable, *K*, produced only minor changes in the output, *H*. The same asymptotic behavior was assessed with the logarithmic fits to the (*K,* *H*) data, with the difference that in this case the fits were obtained by performing a linear regression of *H* against *K* after the logarithmic transformation of *K* alone. In behavioral studies, the fitting parameters can reflect how performance improves to an optimal point before deteriorating, possibly due to fatigue, cognitive overload, or other factors. Accordingly, all parameters for the power law, logarithmic and quadratic fits were tabulated (Table 1) for qualitative comparison across groups to assess if they fit within close tolerance constraints. Given high R^2^ values can sometimes indicate overfitting, visual checks were performed for linearity, homoscedasticity and independence. For linearity we plotted together scatter plots with the fitted lines to visually assess whether the model fits the data well. For homoscedasticity, we plotted the residuals against the fitted values. In these plots, the residuals were randomly scattered with a constant spread, suggesting heteroscedasticity. Independence was assumed due to the pseudorandom presentation of images for the individual picture rating tasks.

**Table 1:** Group fitting parameters for IAPS rating experiment across three cohorts

Curve set	Curve	Cohort					
		EBS		AMHA-1		AMHA-2	
		R^2^	Parameters	R^2^	Parameters	R^2^	Parameters
(log(K),log(H)) (power law)	(log(K_+_), log(H_+_))	0.732	a = 0.440	0.784	a = 0.475	0.767	a = 0.461
			b = 0.296		b = 0.287		b = 0.282
	(log(K_−_), log(H_−_)	0.766	a = 0.473	0.809	a = 0.502	0.827	a = 0.510
			b = 0.274		b = 0.261		b = 0.250
(log(K), H) (logarithmic)	(log(K_+_), H_+_)	0.857	a = 2.210	0.890	a = 2.316	0.875	a = 2.304
			b = 2.047		b = 2.013		b = 1.967
	(log(K_−_), H_−_)	0.871	a = 2.330	0.892	a = 2.324	0.902	a = 2.394
			b = 1.932		b = 1.905		b = 1.851
(K, σ) (quadratic)	(K_+_, σ_+_)	0.750	a = − 0.476	0.746	a = − 0.470	0.812	a =− 0.518
			b = 1.468		b = 1.439		b = 1.588
			c = 0.067		c = 0.091		c = 0.068
	(K_−_, σ_−_)	0.817	a = − 0.553	0.862	a = − 0.580	0.864	a = − 0.609
			b = 1.667		b = 1.754		b = 1.827
			c = 0.065		c = 0.055		c = 0.069

Group power law, (log(*K),* log(*H)*), and logarithmic, (log(*K*)*,* *H*) fits along with quadratic, (*K,* *σ*), fits are listed for the rating data across the three cohorts, stratified by valence of ratings (approach, +; or avoidance, −). For each fit, the coefficient of determination (goodness of fit), *R*^2^, and the corresponding fitting parameters of the group approach (+) and avoidance (*−*) curves is reported per cohort

### (*K, H*) value functions

We evaluated mean positive or negative ratings across stimuli within a picture category (*K*_±_) and the Shannon entropy of these ratings (*H*_±_). We used the following approach to compute the Shannon entropy separately for the positive (approach) and negative (avoidance) ratings in each category. First, consider an ensemble of numbers for either positive or negative rating responses, **a**, across stimuli within a single picture category: **a**_±_  = {a_1_, a_2_,..., a_N_}, for *N* pictures within the given category. We can then define the relative proportions of the positive and negative responses for the individual stimuli, *p*_*i*_, such that

1 $$p_{i} = \frac{{a_{i} }}{{\mathop \sum \nolimits_{j = 1}^{N} a_{j} }}.$$

Using these normalized proportions of the rating responses, the Shannon entropy of the response pattern can be computed for an individual picture category as follows:

2 $$H_{ \pm } = \mathop \sum \limits_{i} p_{i} \log_{2} \left( {\frac{1}{{p_{i} }}} \right).$$

After computing the values of *K*_±_ and *H*_±_ for each picture category, (*K,* *H*) value functions were generated by plotting the Shannon entropy, *H*_±_, against the mean ratings (*K*_±_), for all picture categories for an individual subject. (*K,* *H*) data were also plotted across multiple participants to visualize data at the group level.

At the group level, we assessed if (*K,* *H*) best-fit parameters could be approximated using the logarithmic function, *H*(*K*) = *a* log_10_(*K*) + *b*, or power-law functions, *H*(*K*) = *bK*^*a*^; we also confirmed they contained boundary envelopes that conformed well to either logarithmic functions or power-law functions. At the individual subject level, we assessed fits for the same logarithmic and power-law functions to the (*K,* *H*) data for approach and avoidance across picture categories for individual participants. The best-fit parameters for the logarithmic and power-law functions were achieved by performing a simple linear regression on the plots for *H* versus log_10_(*K*), and log_10_(*H*) versus log_10_(*K*), respectively.

### (*K, H*) limit functions

The second relationship considered was that between the mean ratings, *K*_±_, and the standard deviation of ratings across stimuli within a category, *σ*_±_. (*K,* *σ*) limit functions were generated by plotting values of *σ* against *K* for all picture categories in an individual subject or by pooling the data together across participants in a group analysis. At both the individual and group level, we found that (*K,* *σ*) limit functions were well characterized by quadratic functions of the form *σ* = *aK*^2^ + *bK* + *c*. For the group data, we fit quadratic boundary envelopes to the (*K,* *σ*) data much in the same manner performed for the (*K,* *H*) value functions. Finally, for each subject, quadratic functions were fitted to the (K, σ) data by employing MATLAB® and its polyfit() function (Livengood et al. 2017).

### (*H*_*−*_*, H*_+_) trade-off plots

(H_−_, H_+_) trade-off (or opponency) plots were defined by plotting the Shannon entropy for positive ratings, H_+_, against the Shannon entropy for negative ratings, H_−_, for all picture categories in each stimulus set. These (H_−_, H_+_) plots were generated using either each participant’s data across categories or by pooling data from all participants to generate a group-level plot (Livengood et al. 2017). For both the individual subject and group-level data, (H_−_, H_+_) data conformed to a radial distribution about the origin of the trade-off plot, such that $r=\sqrt{{\left({H}_{+}\right)}^{2}+{\left({H}_{-}\right)}^{2}}$, or equivalently, ${H}_{+}=\sqrt{{r}^{2}-{\left({H}_{-}\right)}^{2}}$. Radial fits were estimated for individual participants as well as the group-level data by computing the mean radial distance, r, across all (H_−_, H_+_) data in the trade-off plot.

### Feature extractions from (*K*,* H*), (*K*,* σ*), and (*H*_−_, *H*_+_) functions

To help characterize the (*K,* *H*), (*K,* *σ*), and (*H*_*−*_*,* *H*_+_) functions, we applied two standard definitions from behavioral economics (*LA* and *RA*) along with the *RA* computation applied to the avoidance arm of the value function (See Fig. 1a), referred to as loss resilience (*LR*) herein. Twelve other features that reflect standard curve feature analyses were utilized (See Fig. 1): positive offset (*β*_+_), negative offset (*β*_*−*_), positive apex (*α*_+_), negative apex (*α*_*−*_), positive turning point (*ρ*_+_), negative turning point (*ρ*_*−*_), positive quadratic area (*q*_+_), negative quadratic area (*q*_*-*_), mean polar angle of the (*H*_*−*_*,* *H*_+_) curve (*θ*), standard deviation of the polar angle (*σ*_*θ*_), mean radial distance of points on the (*H*_*−*_*,* *H*_+_) plot (*r*), and the standard deviation of the radial distances (*σ*_*r*_). These simple metrics are not exhaustive but allow interpretation of the functions based on RPT, prospect theory, and Markowitz’s decision utility (Breiter and Kim 2008; Kahneman and Tversky 1979; Kim et al. 2010; Lee et al. 2015; Livengood et al. 2017; Markowitz 1952; Tversky and Kahneman 1992; Viswanathan et al. 2017). Descriptions of these 15 curve features are provided briefly in what follows, along with their economic behavior term/interpretation. In Table 6 (Appendix), we show each one of the 15 features with their mathematical term, along with their corresponding economic behavioral term and assigned symbol used in tables.

### (K, H) extracted feature definitions

Features for the (*K,* *H*) plots were framed by the (*K,* *H*) function being considered concave relative to the *K*-axis. The RPT features that were extracted from these graphs were: risk aversion (*RA*), loss resilience (*LR*), loss aversion (*LA*), and the positive and negative offsets (*β*_±_).

- Risk aversion (RA): risk aversion is extracted as the ratio of the second derivative of the (K_+_, H_+_) curve to its first derivative, which also produces a curve. To produce a unitary value for comparison across cohorts, we calculated RA for K_+_ = 1.5. Informally, RA measures the degree to which an individual prefers a likely reward in comparison to a better more uncertain reward. RA is a common notion in economics that studies decision-making under uncertainty (Zhang et al. 2014).
- Loss resilience (LR): loss resilience is defined to be the absolute value of the ratio of the second derivative of the (K_−_, H_−_) curve to its first derivative, which also produces a curve. For prediction, we calculated LR at K_−_ = − 1.5. Informally, LR is the degree to which an individual prefers to lose a small defined amount in comparison to losing a greater amount with more uncertainty associated with this loss.
- Loss aversion (LA): loss aversion is the absolute value of the ratio of the linear regression slope of (log K_−_, log H_−_) to the linear regression slope of (log K_+_, log H_+_). It intuitively measures the degree to which an individual person outweighs losses to gains. LA is a fundamental measure in prospect theory (Kahneman and Tversky 1979), which informally states that humans have a cognitive bias to overweight losses relative to gains in the presence of uncertainty.
- Positive offset (β_+_) or Ante: the positive offset is the value of K_+_ when setting H_+_ = 0. β_+_ intuitively measures the ante one needs to engage in a game of chance and models the amount of a bid an individual is willing to make to enter a game of chance (e.g., an “ante” in poker).
- Negative offset (β_−_) or Insurance: the negative offset is the value of K_−_ when setting H_−_ = 0. β_−_ intuitively measures how much insurance an individual might need against bad outcomes. It mirrors the “ante”, but in the framework of potential losses.

### (K, σ) extracted feature definitions

Features for the (*K,* *σ*) curves, were framed by the (*K,* *σ*) curves being considered as quadratic functions, which were concave relative to the *K*-axis. The (*K,* *σ*) curve models the relationship between variance (risk) and mean value. It can also be framed by the following question: Would an individual prefer a dollar with probability one, or a value drawn from a normal distribution with a mean of two and variance of two? The RPT features that are extracted from this curve include: the positive and negative apices (*α*_±_), the positive and negative turning points (*ρ*_±_), and the positive and negative quadratic areas (*q*_±_).

- Positive apex (α_+_) or Peak Positive Risk: the positive apex is the value of σ_+_ for the derivative $\frac{d{\sigma }_{+}}{d{K}_{+}}=0$. Intuitively, this represents the maximum variance for approach behavior. In this sense, α_+_ models where increases in positive value transition from a relationship with increases in risk, to a relationship with decreases in risk. Markowitz (1952) described decision utility similarly, so that the positive apex models when variance changes from weighing against a decision to facilitating a decision.
- Negative apex (α_−_) or Peak Negative Risk: the negative apex is the value of σ_-_ when the derivative $\frac{d{\sigma }_{-}}{d{K}_{-}}=0$. Intuitively, this represents the maximum variance for avoidance behavior. Like with the positive apex, this transition point is important to consider for avoidance decisions in the context of decision utility by Markowitz (1952).
- Positive turning point (ρ_+_) or Reward Tipping Point: the positive turning point is the value of K_+_ when the derivative $\frac{d{\sigma }_{+}}{d{K}_{+}}=0$. Intuitively, this represents the rating intensity with maximum variance for approach behavior, potentially when an individual decides to approach a goal-object.
- Negative turning point (ρ_−_) or Aversion Tipping Point: the negative turning point is the value of K_−_ when the derivative $\frac{d{\sigma }_{-}}{d{K}_{-}}=0$. Intuitively, this represents the rating intensity with maximum variance for avoidance behavior, potentially when an individual decides to avoid a goal-object.
- Positive quadratic area (q_+_) or Total Reward Risk: the positive quadratic area is the area under the curve (AUC) of the first quadrant of the (K_+_, σ_+_). This variable represents the relationship between K_+_ and σ_+_ and can be thought of as a quantity that measures the amount of value an individual associates to positive stimuli.
- Negative quadratic area (q_−_) or Total Aversion Risk: the negative quadratic area is the AUC of the third quadrant for the curve (K_−_, σ). This variable represents the relationship between K_−_ and σ and can be thought of as quantity that measures the aversive value an individual associates to negative stimuli.

### (H_−_, H_+_) extracted feature definitions

Features for the (*H*_*−*_*,* *H*_+_) curve were framed as the (*H*_*−*_*,* *H*_+_) function being considered a trade-off function between the *H*_*−*_ and *H*_+_ variables, which can commonly look like a semi-circular fit in individuals (e.g., See Fig. 1c). The RPT features extracted from this curve include: the mean polar angle (*θ*), its standard deviation (*σ*_*θ*_), mean radial distance (*r*), and its corresponding standard deviation (*σ*_*r*_).

- Mean polar angle (θ) or Reward-Aversion Tradeoff: the mean polar angle is the mean of the polar angles of the points in the (H_−_, H_+_) plane. Intuitively, this measures the mean balance for the entropies, or patterns, in approach vs. avoidance behavior. It signifies the balance in approach and avoidance judgments across multiple categories of goal-object (e.g., picture ensembles in this case).
- Polar angle standard deviation (σ_θ_) or Tradeoff Range: the standard deviation of the polar angles of the points in the (H_−_, H_+_) plane. Intuitively, this measures the standard deviation in the patterns of approach and avoidance behavior. This variance represents the spread of positive and negative preferences across a set of potential goal-objects and can be considered a measure of the breadth of an individual’s (or a group’s) portfolio of preference.
- Mean radial distance (r) or Reward-Aversion Consistency: the mean radial distance measures the average Euclidean distance of the data points in the (H_−_, H_+_) curve to the origin. This measure defines how individuals can have strong positive and negative preferences (i.e., biases) for the same thing, reflecting conflict, or have low positive and negative preferences for something, reflecting indifference. This gets at the consistency of compatibility of approach and avoidance, and how an individual can both like and dislike something or be indifferent to both its positive and negative features.
- Radial distance standard deviation (σ_r_) or Consistency Range: this is simply the standard deviation of the radial distances of the data points in the (H_−_, H_+_) plane to the origin. This final measure is interpreted through how the points in the (H_−_, H_+_) plane vary regarding the radial distance from the origin. The variance in this radial distance will reflect how much an individual goes between having conflicting preferences and having indifferent ones.

### Comparison of features between rating experiments

For each of the three subject populations, the mean and standard deviation (SD) were computed for each of the fifteen features, along with standard error of the mean (SEM) and the 95% confidence intervals (CI) for the corresponding means. Violin plots (Hintze and Nelson 1998) for each of the RPT features were also generated to provide a visual comparison of the distribution, interquartile range (IQR), and 95% CIs, with respect to the corresponding median, for each RPT feature across all cohorts. The primary framework of comparison was assessment of overlap in the 95% CI for the corresponding means, and violin plots for the medians.

A quantitative comparison of RPT features across the three cohorts was also performed using rank-based, nonparametric Kruskal–Wallis *H*-test (“Kruskal–Wallis Test,” 2008b) statistics, followed by post-hoc nonparametric pairwise multiple comparisons using Dunn’s test(Dinno 2015) and Kolmogorov–Smirnov test (“Kolmogorov–Smirnov Test,” 2008a) statistics. This was done for all fifteen features, although only seven of these features reflected dimensionless units, and covariances around demographic differences in the cohorts could not be incorporated into such analyses. Given this last factor, we assessed age distributions for each sample, where clear skewing existed, and ran univariate linear regressions for the fifteen features against age in that cohort to assist with interpreting results.

## Results

### Group-level assessment of positive and negative ratings by picture category

For each of the three groups of participants studied, we summed the total number of positive and negative ratings made per picture category, and their mean, as a qualitative assessment that there were no major deviations in IAPS stimuli ratings across the three studies. As can be seen in Table 7 (Appendix), the mean positive and negative ratings for each category of picture were in close alignment across the EBS, AMHA-1 and AMHA-2 groups.

### Group-level (*K*,* H*), (*K*,* σ*), and (*H*_−_, *H*_+_) analyses

We first investigated the relationships between mean ratings and the Shannon entropy of category distributions for ratings. Group-level analyses were performed in two ways:

1. Envelope fits as done previously (Kim et al. 2010; Livengood et al. 2017) and
2. Statistical fits of group data to constrain the fits tested subsequently with individual data (Table 1).

For envelope fitting of the value function, power-law and logarithmic boundary envelopes were fit to the approach (*K*_+_*,* *H*_+_) and avoidance (*K*_*−*_*,* *H*_*−*_) rating data such that they formed an outer bound containing 95% of the data; they were both observed to provide robust approximations of the edge of the distribution. For all three experiments, group data was fit by boundary envelopes to similar extents (all *p* < 0*.*05) by both logarithmic and power-law functions. When we examined functional fitting of the group data, we observed statistically significant fits (all *p* < 0*.*05) by both logarithmic and power-law functions (Table 1). *R*^2^ values were all > 0*.*70 and extended up to 0*.*90 across the three cohorts. Given high R2 values, visual checks were performed for linearity and homoscedasticity. Scatter plots with the fitted lines showed the model fit the data well. Residuals were plotted against the fitted values and showed the residuals were randomly scattered with a constant spread, supporting heteroscedasticity.

Next, we examined the relationship between the average category ratings and the standard deviation of ratings in each category, which we refer to as the mean–variance relationship (See Fig. 1b). Boundary envelopes enclosing 95% of the approach (*K*_+_*,* *σ*_+_) and avoidance (*K*_*−*_*,* *σ*_*−*_) data were fit to the EBS data. The quadratic boundary envelopes effectively approximated the edge of the mean–variance plots. The same analytic approach was performed for AMHA-1 and AMHA-2 data, showing outer bounds containing 95% of the data, providing robust approximations of the edge of the distribution, and broadly corroborating the behavior of analogous distributions for reported keypress-based RPT variables (Kim et al. 2010; Livengood et al. 2017), with *p* < 0*.*05 for all three datasets. When we examined functional fitting of the group data, we observed statistically significant fits (all *p* < 0*.*05) for all three cohorts (Table 1). Importantly, all curves depicting limit functions with group data showed concave fits (relative to the absolute value of the {K, H, σ} variables), thereby setting a constraint used for the individual data. *R*^2^ values for group fitting varied from 0.732 to 0.902 across the three cohorts (Table 1).

Lastly, we examined the (*H*_*−*_*,* *H*_+_) trade-off distributions characterizing the relationship between the patterns of approach and avoidance across tasks. This analysis sought to assess whether the pattern of approach preference behavior (i.e., positive ratings) scaled in proportion to the avoidance preference behavior (i.e., negative ratings) for pictures within the same categories. Specifically, we fit radial functions to test for symmetry in the distribution of *H*_*−*_ and *H*_+_ values across categories within each individual subject. Ratings-based group-level (*H*_*−*_*,* *H*_+_) distributions were broader than distributions we have reported previously for keypress-based implementations of the IAPS relative preference task, consistent with the increased variance in the rating graph features for mean polar angle and mean radial distance.

### Individual subject (*K, H*) value functions

**Fig. 2:**
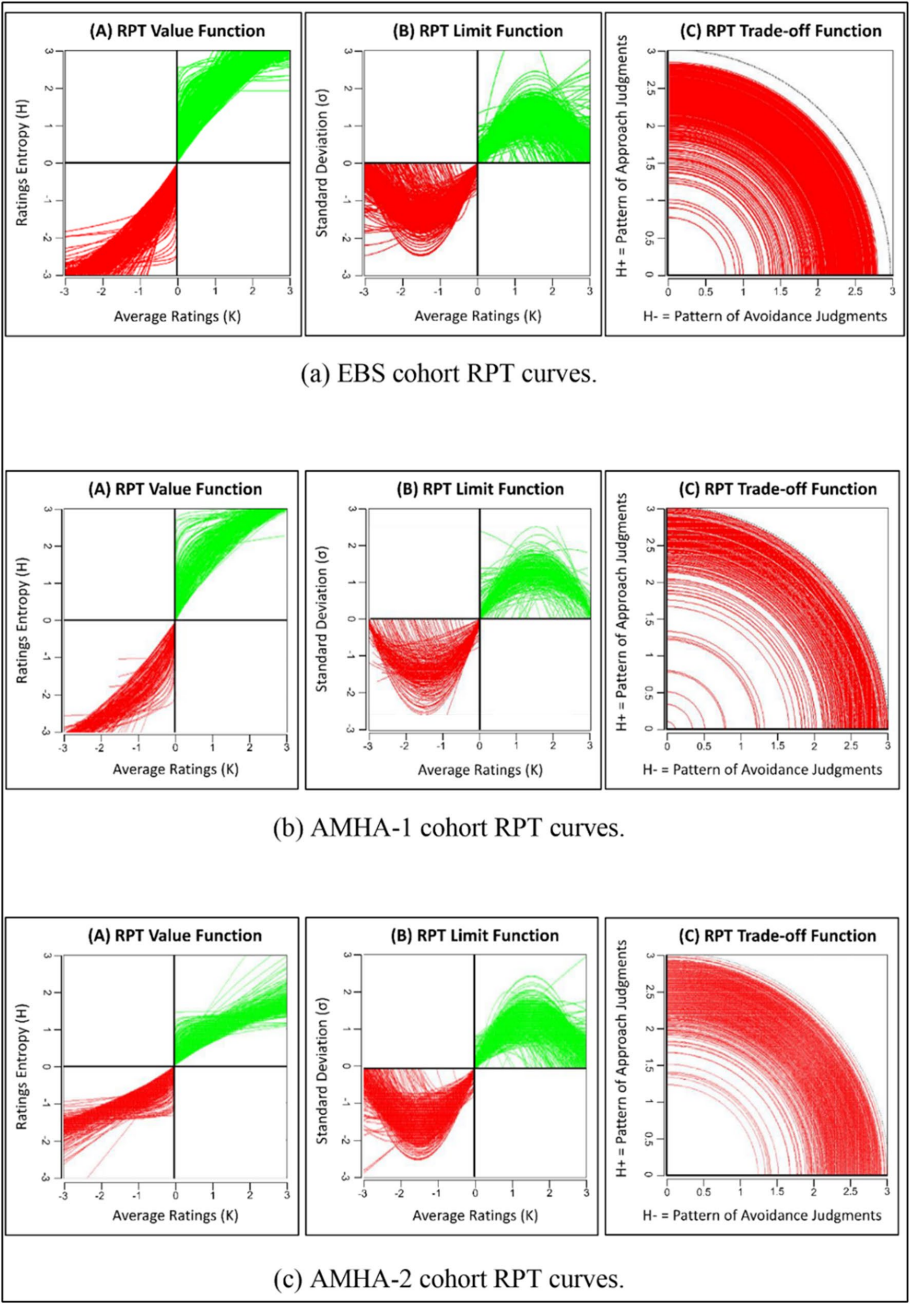
Individual RPT fits of the three distinct cohorts for the IAPS picture rating task. **A** Value functions comparing average rating intensity (*K*) to rating entropy (*H*) in individual participants, where *K* and *H* values were computed across the six picture categories, for either approach (*K*_+_*,* *H*_+_) or avoidance (*K*_*−*_*,* *H*_*−*_) rating behavior within a single representative subject. Green and red traces indicate power-law fits to approach and avoidance data for each subject. **B** Limit functions comparing *K* to the standard deviation of approach or avoidance ratings (*σ*) across picture categories in individual participants. Approach and avoidance data for individual participants were fit to quadratic functions (see Methods). **C** Trade-off plot comparing entropy for approach (*H*_+_) and avoidance (*H*_*−*_) ratings across six picture categories in individual participants. The dotted black line denotes *r* = log_2_(8) and each subject is shown as a radial fit where $r=\sqrt{{\left({H}_{+}\right)}^{2}+{\left({H}_{-}\right)}^{2}}$

After investigating the shape of distributions at the group level, we assessed (*K,* *H*) data and value function fitting at the individual subject level. As observed for group data, individual participants’ (*K,* *H*) value functions for the rating were well fit by concave logarithmic or power-law functions (See Fig. 2 and Table 2). Goodness of fit was assessed by computing *R*^2^ values, adjusted *R*^2^ values (accounting for degrees of freedom), and $F$-statistics for each subject’s model fit (Table 2). *R*^2^ values were all > 0*.*80 and ranged from 0.84 to 0.96. Average goodness of fit values were quite similar between the three cohorts (Table 2).

**Table 2:** Goodness of fit summary statistics for individual value and limit functions

Curve set	Curve	Summary statistic	Mean ± Std. dev. (per cohort)		
			EBS	AMHA-1	AMHA-2
(log(K), log(H)) (power law)	(log(K_+_), log(H_+_))	R^2^	0.84 ± 0.20	0.86 ± 0.17	0.89 ± 0.17
		R^2^_adj_	0.76 ± 0.32	0.80 ± 0.26	0.80 ± 0.29
		F-value	2.21 × 10^29^ ± 2.20 × 10^30^	1.10 × 10^30^ ± 1.39 × 10^31^	3.43 × 10^29^ ± 6.57 × 10^30^
	(log(K_−_), log(H_−_)	R^2^	0.92 ± 0.12	0.91 ± 0.14	0.95 ± 0.10
		R^2^_adj_	0.87 ± 0.21	0.85 ± 0.24	0.90 ± 0.20
		F-value	1.40 × 10^30^ ± 1.26 × 10^31^	7.15 × 10^29^ ± 4.59 × 10^30^	1.14 × 10^30^ ± 8.26 × 10^30^
(log(K), H) (logarithmic)	(log(K_+_), H_+_)	R^2^	0.89 ± 0.14	0.89 ± 0.16	0.92 ± 0.13
		R^2^_adj_	0.85 ± 0.21	0.85 ± 0.23	0.87 ± 0.21
		F-value	1.23 × 10^30^ ± 1.01 × 10^31^	8.00 × 10^29^ ± 1.24 × 10^31^	1.90 × 10^30^ ± 2.06 × 10^31^
	(log(K_−_), H_−_)	R^2^	0.94 ± 0.11	0.92 ± 0.12	0.96 ± 0.07
		R^2^_adj_	0.90 ± 0.18	0.88 ± 0.21	0.80 ± 0.29
		F-value	2.44 × 10^30^ ± 2.21 × 10^31^	1.85 × 10^30^ ± 1.43 × 10^31^	3.61 × 10^30^ ± 2.34 × 10^31^
(K, σ) (quadratic)	(K_+_, σ_+_)	R^2^	0.87 ± 0.18	0.85 ± 0.19	0.90 ± 0.13
		R^2^_adj_	0.78 ± 0.30	0.75 ± 0.31	0.84 ± 0.21
		F-value	1.24 × 10^30^ ± 1.52 × 10^31^	2.33 × 10^32^ ± 3.95 × 10^33^	1.12 × 10^30^ ± 1.50 × 10^31^
	(K_−_, σ_−_)	R^2^	0.93 ± 0.11	0.93 ± 0.10	0.95 ± 0.10
		R^2^_adj_	0.88 ± 0.18	0.89 ± 0.17	0.90 ± 0.17
		F-value	1.34 × 10^31^ ± 1.52 × 10^32^	6.93 × 10^31^ ± 9.46 × 10^32^	2.26 × 10^30^ ± 5.00 × 10^31^

Individual logarithmic, (log(*K*)*,* *H*), and linear (log(*K*)*,* log(*H*)) fits, along with quadratic, (*K,* *σ*), fits are listed for the rating data across the three cohorts. Linear, logarithmic, and quadratic correlations were performed in each subject across the data relating to approach ratings for the six categories of IAPS stimuli (aggressive animals, nature, cute animals, disaster, people in bathing suits and sports), and across the data relating to avoidance responses; participants needed data from at least three of the experimental conditions to be fitted. The mean and standard deviation are listed for the coefficient of determination, *R*^2^ and its corresponding adjusted value R^2^_adj_

Overall, fewer participant exclusions were noted across the three studies for logarithmic fitting of the (*K,* *H*) approach data when compared to (*K,* *H*) power-law model fits due to either insufficient data for fitting of joint RPT distributions or invalid parameter estimates (see Methods).

### Individual subject (*K, σ*) limit functions

The consistency of mean–variance relationships across participants in the rating experiment was assessed by fitting quadratic functions to each individual subject’s (*K,* *σ*) distributions for approach and avoidance rating data. Concave quadratic fits across individual participants’ (*K,* *σ*) data are displayed in Fig. 2. As with the (*K,* *H*) data, goodness of fit was assessed by computing *R*^2^ values, adjusted *R*^2^ values (accounting for degrees of freedom), and $F$-statistics for each subject’s model fit (Table 2). *R*^2^ values varied from 0.85 to 0.95, which was considered very high across participants.

### Individual subject (*H*_*−*_*, H*_+_) trade-off functions

Trade-off distributions (*H*_*−*_*,* *H*_+_) for individual participants’ rating patterns across pictures were also examined. This analysis sought to assess whether the pattern of participants’ approach preference behavior (i.e., positive ratings) scaled in proportion to the pattern of participants’ avoidance preference behavior (i.e., negative ratings) for pictures within the same categories (e.g., nature scenes). Specifically, we fit radial functions to test for trade-offs in the distribution of *H*_*−*_ and *H*_+_ values across categories within each individual subject. Figure 2 displays radial fits across individual participants’ (*H*_*−*_*,* *H*_+_) data and highlight the (*H*_*−*_*,* *H*_+_) data points and fit for a representative subject from each experiment.

### Extracted curve features computation and comparison of rating results

**Fig. 3:**
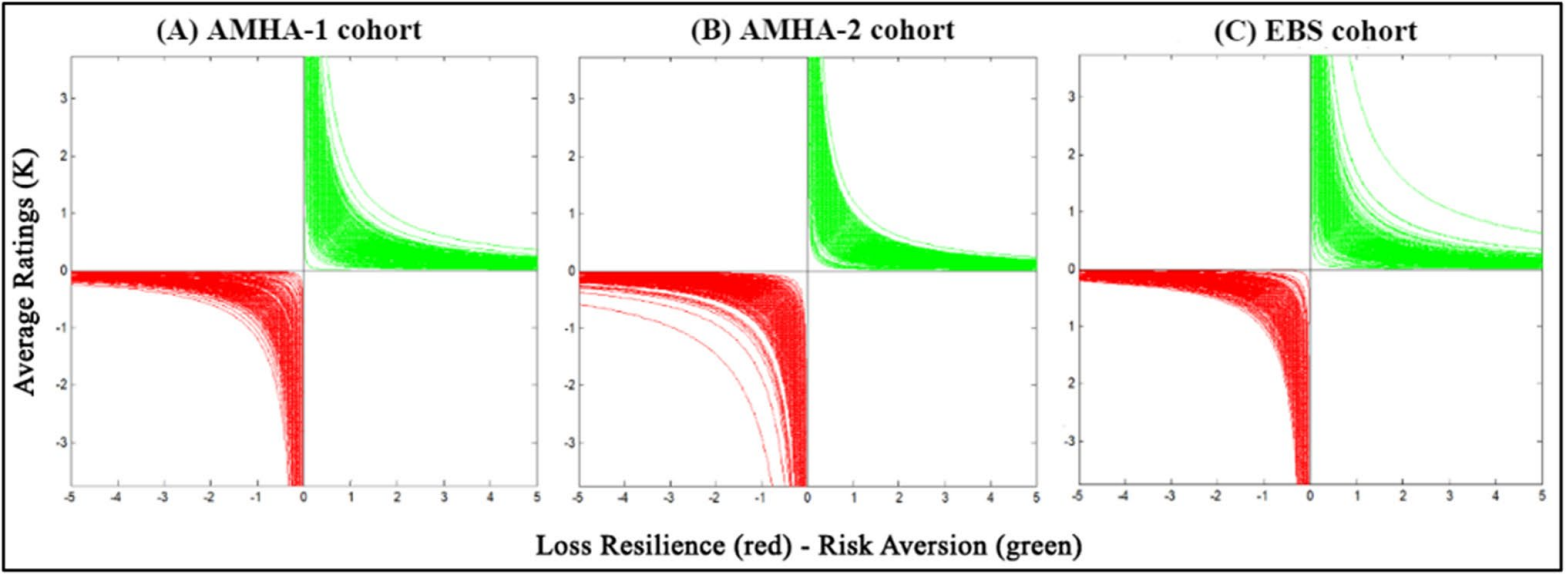
Individual risk aversion (*RA*) and loss resilience (*LR*) functions for the three cohorts. **A** Risk aversion (*RA*) functions comparing computed *RA* to average picture ratings (*K*) in individual participants are shown for the AMHA-1 cohort. Loss resilience (*LR)* to average picture ratings intensity (*K*) in individual participants are shown as well. **B** Risk aversion functions and loss resilience functions shown for the AMHA-2 cohort. **C** Risk aversion functions and loss resilience functions show for the EBS cohort. Note the hyperbolic functional forms in green (approach) and red (avoidance) for each curve

**Fig. 4:**
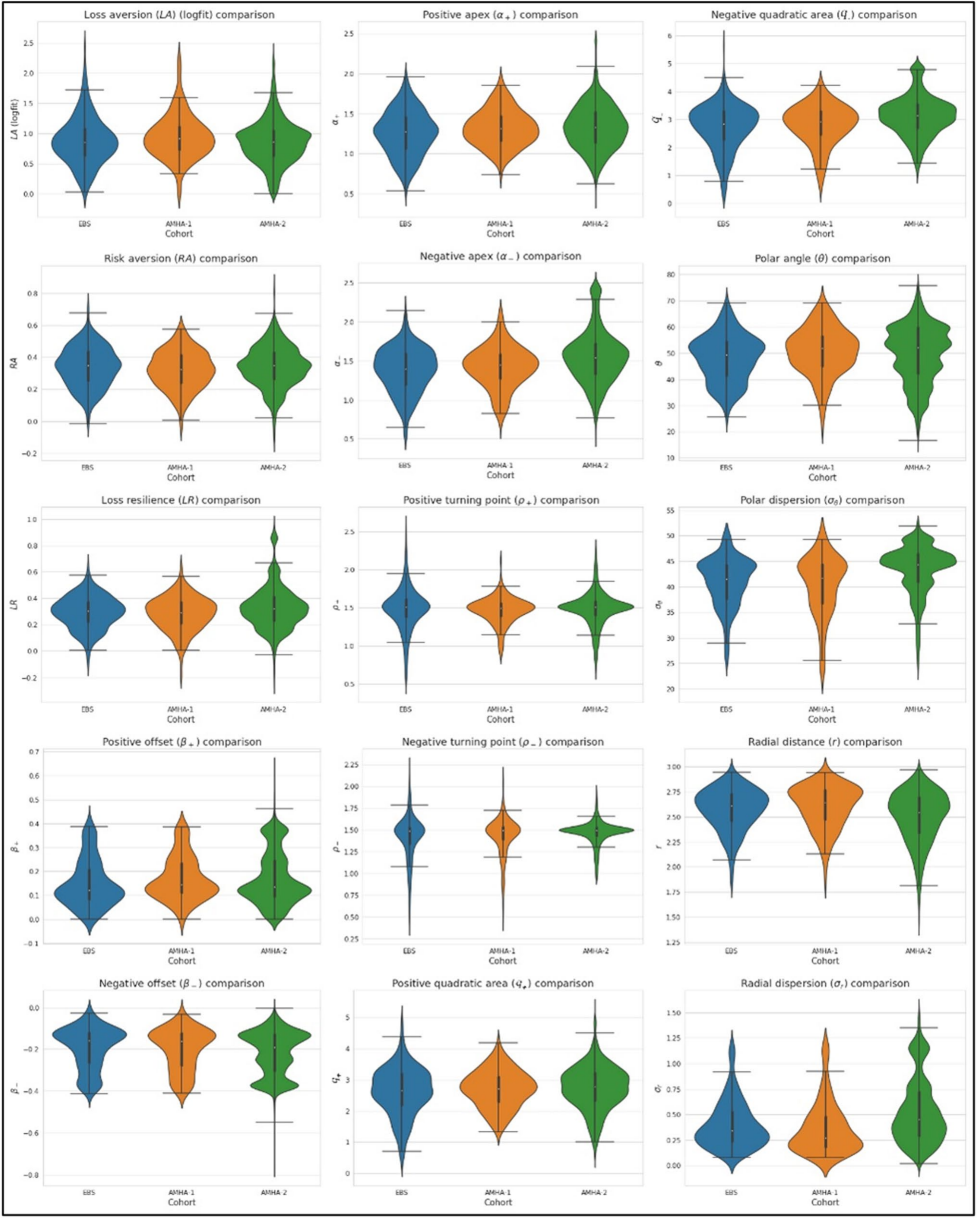
Violin plots for each of the RPT features are tiled to provide a hybrid visual comparison of the distribution, interquartile range (IQR), and 95% CIs, with respect to the corresponding median, for each RPT feature across all cohorts. Kernel density estimates show the shapes of individual distributions for each cohort, while the box plots within each violin plot describes the median and the corresponding IQRs and 95% CIs for the median. Unlike violin plots, box plots don’t allow us to see variations in the data, particularly for multimodal distributions (those with multiple peaks)

**Fig. 5:**
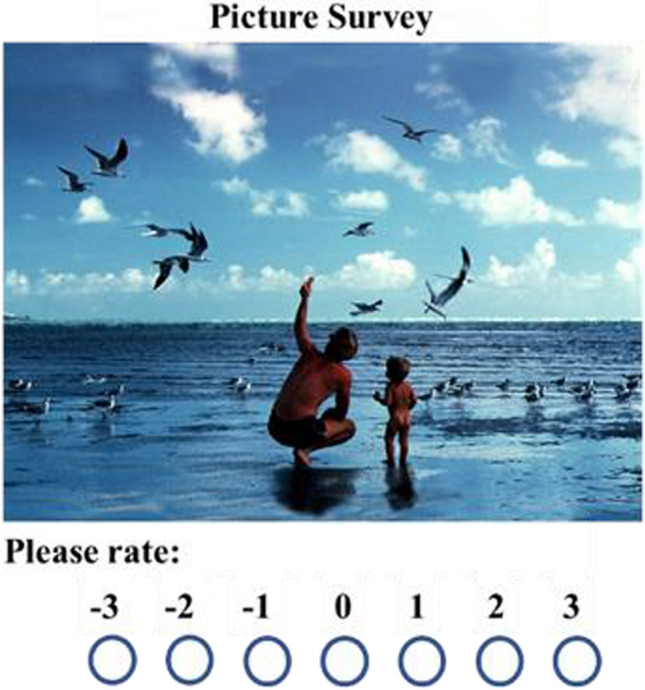
Example of the format of the picture rating task. Unlike the keypress task from prior studies, this task involved no operant consequence to its action. Individuals made ratings with no change in viewing time or other consequence, rating along a 7-point Likert-like scale from − 3 to + 3

Summary statistics for *LA*, *RA*, and the 13 other RPT graph features obtained from each participant in each experiment are summarized in Table 3. The graphical results for RA and LR are shown across the three cohorts in Fig. 3, showing qualitative similarity. Significantly, there was a consistent overlap among the 95% CIs for the median in the majority of the RPT features (See Fig. 4). As is clear in Fig. 4, the majority of RPT features’ distributions showed significant deviation from normality in each cohort, so further analyses used nonparametric statistics, which are tabulated in Table 4. Nonparametric comparison of all the RPT metrics across the three datasets were performed, using the Kruskal–Wallis H test and the corresponding post-hoc pairwise Dunn’s test with Holm-Bonferroni p-value correction and two-sample Kolmogorov–Smirnov (K-S) test. *LA* values were lower in all three cohorts than has been reported for keypress experiments and prospect theory-based experiments in references (Tom et al. 2007; Tversky and Kahneman 1992). It should be noted that *LA* << 2*.*0 for the rating experiments suggests that participants did not show *LA* per se, but potential reward sensitivity. *LA* values did differ between the three cohorts by the Kruskal–Wallis *H*-test, while the post-hoc Dunn’s assessment confirmed that this difference only occurred between the AMHA-1 and AMHA-2 cohorts. The additional post-hoc two-sample K-S test assessment concluded that the distributions for *LA* across the three cohorts did not differ. The results for comparing *RA* across subjects using a defined point on these functions is shown in Table 4. These results demonstrate that *RA* distributions across all three cohorts did not differ statistically using the Kruskal–Wallis *H*-test, as well as the post-hoc Dunn’s test and two-sample K-S test assessments.

**Table 3:** RPT curve metrics for IAPS rating experiments across cohorts

RPT metric	Statistic	Cohort		
		EBS	AMHA-1	AMHA-2
Loss aversion (*LA*)	Mean ± SD	0.88 ± 1.148	1.51 ± 2.090	0.88 ± 0.365
	SEM	0.0738	0.1647	0.0063
	95% CI	[0.74, 1.03]	[1.19, 1.84]	[0.86, 0.89]
Risk aversion (*RA*)	Mean ± SD	0.35 ± 0.122	0.32 ± 0.120	0.34 ± 0.125
	SEM	0.0072	0.009	0.0022
	95% CI	[0.33, 0.36]	[0.31, 0.34]	[0.34, 0.35]
Loss resilience (*LR*)	Mean ± SD	0.32 ± 0.122	0.30 ± 0.130	0.32 ± 0.134
	SEM	0.0073	0.0098	0.0024
	95% CI	[0.29, 0.32]	[0.28, 0.31]	[0.32, 0.33]
Positive offset (*β*_+_)*	Mean ± SD	0.15 ± 0.101	0.16 ± 0.097	0.17 ± 0.106
	SEM	0.0046	0.0052	0.0018
	95% CI	[0.14, 0.16]	[0.15, 0.17]	[0.17, 0.18]
Negative offset (*β*_*-*_)*	Mean ± SD	− 0.19 ± 0.101	− 0.19 ± 0.101	− 0.21 ± 0.108
	SEM	0.0048	0.0061	0.0018
	95% CI	[− 0.20, − 0.18]	[− 0.20, − 0.18]	[− 0.21, − 0.21]
Positive apex (*α*_+_)*	Mean ± SD	1.27 ± 0.327	1.27 ± 0.274	1.33 ± 0.290
	SEM	0.0154	0.0152	0.0049
	95% CI	[1.24, 1.30]	[1.24, 1.30]	[1.32, 1.34]
Negative apex (*α*_+_)*	Mean ± SD	1.39 ± 0.378	1.40 ± 0.382	1.52 ± 0.349
	SEM	0.0174	0.0216	0.0059
	95% CI	[1.35, 1.42]	[1.35, 1.44]	[1.51, 1.53]
Positive turning point (*ρ*_+_)*	Mean ± SD	1.48 ± 0.269	1.48 ± 0.203	1.48 ± 0.195
	SEM	0.0131	0.0116	0.0034
	95% CI	[1.45, 1.50]	[1.45, 1.50]	[1.47, 1.48]
Negative turning point (*ρ*_*-*_)*	Mean ± SD	1.40 ± 0.275	1.38 ± 0.301	1.48 ± 0.103
	SEM	0.0131	0.0176	0.0019
	95% CI	[1.37, 1.42]	[1.34, 1.41]	[1.48, 1.49]
Positive quadratic area (*q*_+_)*	Mean ± SD	2.63 ± 0.867	2.59 ± 0.783	2.71 ± 0.759
	SEM	0.0412	0.0437	0.013
	95% CI	[2.55, 2.71]	[2.51, 2.68]	[2.68, 2.73]
Negative quadratic area (*q*_*-*_)*	Mean ± SD	2.68 ± 1.031	2.66 ± 1.028	3.05 ± 0.815
	SEM	0.0477	0.0583	0.0139
	95% CI	[2.58, 2.77]	[2.55, 2.78]	[3.02, 3.08]
Polar angle (*θ*)	Mean ± SD	52.53 ± 14.654	58.89 ± 16.927	50.04 ± 11.499
	SEM	0.656	0.8872	0.195
	95% CI	[51.24, 53.82]	[57.14, 60.63]	[49.66, 50.42]
Polar dispersion (*σ*_*θ*_)	Mean ± SD	40.73 ± 7.024	34.40 ± 15.146	40.73 ± 7.024
	SEM	0.3203	0.7917	0.3203
	95% CI	[40.10, 41.36]	[32.84, 35.95]	[40.10, 41.36]
Radial distance (*r*)	Mean ± SD	2.52 ± 0.256	2.60 ± 0.239	2.52 ± 0.256
	SEM	0.3203	0.7917	0.3203
	95% CI	[2.50, 2.55]	[2.57, 2.62]	[2.50, 2.55]
Radial dispersion (*σ*_*r*_)	Mean ± SD	0.47 ± 0.298	0.38 ± 0.276	0.47 ± 0.298
	SEM	0.0133	0.0145	0.0133
	95% CI	[0.44, 0.49]	[0.35, 0.40]	[0.44, 0.49]

RPT features of the (*K,* *H*), (*K,* *σ*), and (H_−_, H_+_) curves of the IAPS picture rating data across the three distinct cohorts. Fifteen features were identified using common engineering methods, including five features from the value function, six from the limit function, and four from the trade-off function (see Methods). For each of the three datasets, the mean and standard deviation (SD) are listed for the fifteen features, along with standard error of the mean (SEM) and the 95% confidence intervals (CI) for the corresponding means. Unstarred features of the (*K,* *H*), (*K,* *σ*), and (H_−_, H_+_) curves are in dimensionless units, facilitating comparison across cohorts

**Table 4:** Nonparametric comparison of RPT metrics across distinct cohorts for IAPS picture rating experiment and corresponding post-hoc pairwise Dunn’s test and two-sample Kolmogorov–Smirnov (K-S) test statistics

RPT metric	Kruskal–Wallis H-test		Dunn’s test *p*-values (Holm-Bonferroni corrected)			Two-sample K-S test *p*-values		
	H-value	*p*-value	AMHA-1/AMHA-2	AMHA-1/EBS	EBS/AMHA-2	AMHA-1/AMHA-2	AMHA-1/EBS	EBS/AMHA-2
Loss aversion (*LA*) (logfit)	6.98785	3.0381 × 10^−2^	0.025	0.097	0.8	0.051	0.15	0.63
Risk aversion (*RA*)	4.07003	1.3068 × 10^−1^	0.13	0.29	0.79	0.052	0.14	0.79
Loss resilience (*LR*)	13.3089	1.2883 × 10^−3^	0.019	0.59	0.019	0.017	0.84	0.027
Positive offset (*β*_+_)*	10.2435	5.9655 × 10^−3^	0.4	0.017	7.50 × 10^−3^	0.19	8.90 × 10^−4^	0.001
Negative offset (*β*_*-*_)*	20.1216	4.2722 × 10^−5^	0.078	0.33	1.20 × 10^−4^	0.077	0.5	1.10 × 10^−3^
Positive apex (*α*_+_)*	14.3479	7.6627 × 10^−4^	0.58	0.094	4.80 × 10^−4^	0.27	0.015	1.70 × 10^−3^
Negative apex (*α*_+_)*	74.5914	6.3486 × 10^−17^	0.400 × 10^−6^	0.32	2.00 × 10^−13^	4.300 × 10^−6^	0.36	1.10 × 10^−9^
Positive turning point (*ρ*_+_)*	2.28294	3.1935 × 10^−1^	0.42	0.42	0.82	0.099	0.057	0.49
Negative turning point (*ρ*_*-*_)*	6.16587	4.5824 × 10^−2^	0.67	0.67	0.057	0.061	0.62	2.90 × 10^−4^
Positive quadratic area (*q*_+_)*	5.38677	6.7652 × 10^−2^	0.79	0.79	0.082	0.38	0.41	0.073
Negative quadratic area (*q*_*-*_)*)	65.4338	6.1835 × 10^−15^	1.10 × 10^−5^	0.41	9.60 × 10^−12^	3.60 × 10^−4^	0.64	1.60 × 10^−7^
Polar angle (*θ*)	9.9913	6.7672 × 10^−3^	0.34	0.015	9.60 × 10^−3^	0.018	0.026	5.30 × 10^−6^
Polar dispersion (*σ*_*θ*_)	125.001	7.1836 × 10^−28^	6.00 × 10^−13^	0.89	1.40 × 10^−18^	2.10 × 10^−11^	0.56	3.80 × 10^−15^
Radial distance (*r*)	40.4942	1.6099 × 10^−9^	1.500 × 10^−6^	0.18	5.10 × 10^−5^	1.30 × 10^−5^	0.055	2.90 × 10^−4^
Radial dispersion (*σ*_*r*_)	89.6117	3.4759 × 10^−20^	9.600 × 10^−13^	0.09	1.70 × 10^−10^	1.10 × 10^−11^	8.10 × 10^−4^	1.60 × 10^−9^

Non-parametric statistics comparing the fifteen RPT features listed in Table 3 across the three cohorts for the IAPS rating task experiment. Provided are the Kruskal–Wallis H-test scores and their corresponding *p*-values for a three-way comparison in determining statistically significant differences in the distributions across the three rating task cohorts. Given at least 5 observations for each group, of three total, we can approximate the critical values regarding the null hypothesis using the same critical values as a χ^2^-test, given two degrees of freedom for this comparison (number of groups minus 1). As a post-hoc assessment, this table also contains the *p*-values from a pairwise comparison between cohorts using Dunn’s test (*p*-values are corrected using the Holm-Bonferroni method), as well as *p*-values from multiple pairwise comparisons of the distributions for each RPT metric across the cohorts using two-sample Kolmogorov–Smirnov (K–S) non-parametric tests. Unstarred features of the (K, H), (K, σ), and (H−, H+) curves are in dimensionless units, facilitating comparison across cohorts

Loss resilience (*LR*), which is computed the same way as *RA* from the approach curve, but in this case using the avoidance curve, represents the function shown in the lower parts of the graphs in Fig. 3. The three rating experiments produced similar functional forms for *LR* curves; it should be noted that the *LR* and *RA* curves with the rating experiment were similar in means and contained overlapping confidence intervals for the mean, as shown in Table 3. Additionally, there was statistically significant difference in *LR* across the three cohorts according to Table 4, however the post-hoc assessments both highlight that differences in *LR* for the AMHA-1 and EBS cohorts were not statistically significant (*p* > 0*.*05).

Positive and negative offsets, *β*_+_ and *β*_*−*_ respectively, are clearly present in the logarithmic fits to the value function across the three rating experiments. For comparison they were computed from the logarithmic fit and did not significantly differ across cohorts. It should be noted that prospect theory does not allow for offsets to the value function and sets an inflection point connecting the positive and negative arms of the value function so there cannot be offsets for both functions (Kahneman and Tversky 1979; Tversky and Kahneman 1992). The current data confirms prior findings using an analysis of RPT features showing the existence of clear offsets to the value function from the origin when using (*K,* *H*) variables, and a discontinuity along the *K*-axis intercepts between the approach and avoidance data(Kim et al. 2010; Livengood et al. 2017; Viswanathan et al. 2017). Although the Kruskal–Wallis *H*-test results in Table 4 show statistical significance in both *β*_+_ and *β*_*−*_, the post-hoc assessments in Table 4 don’t demonstrate statistically significant differences in the distributions between AMHA-1 and AMHA-2 for *β*_+_ and *β*_*−*_, and AMHA-1 and EBS for just *β*_*−*_.

Apex (*α*_±_), turning point (*ρ*_±_), and quadratic area (*q*_±_) features are standard metrics of parabolic fits for the limit function (i.e., the fit for the mean–variance curves). The apices (*α*_±_) significantly differed across the three cohorts, however a number of the post-hoc assessments do not depict statistically significant differences (see Table 4). For the positive turning point, *ρ*_+_, there was not a statistically significant difference across all cohorts (see Table 4), however the negative turning point, *ρ*_*−*_, statistically differed in the Kruskal–Wallis three-way comparison. The post-hoc assessments for *ρ*_*−*_ shows that differences in the distributions were not statistically significant except for the K-S test’s results in comparing the AMHA-2 and EBS cohorts; in contrast to these observations, Fig. 4 shows overlapping 95% CIs for the median. For the positive quadratic area, *q*_+_, there were not statistically significant differences in approach and avoidance (Table 4), however for *q*_*−*_ the Kruskal–Wallis comparison (Table 4) and post-hoc assessments indicate no statistical significance in differences for just the AMHA-1 and EBS cohorts. Qualitatively, the limit curves for approach and avoidance appear symmetric to each other relative to the *H*-axis (information/value) for the rating experiment; this is not the case for published keypress data (Kim et al. 2010; Lee et al. 2015; Livengood et al. 2017; Viswanathan et al. 2017).

Trade-off curve features showed consistent statistical differences for all four features across the three cohorts for the three-way Kruskal–Wallis comparison (Table 4), as well as non-overlapping CIs for the mean (Table 3). The mean polar angle, *θ*, for the rating experiments was > 45°, consistent with a slight weighting of the rating assessments toward approach. The consistency between approach and avoidance variables is encoded in the radial distance feature, *r*, (i.e., if the increases in approach balanced decreases in avoidance, or if an individual felt more conflict, namely increases in both approach and avoidance). The radial distance features for all three experiments were just slightly within the semicircle described for *r* = log_2_(8), a theoretical frame for ensembles of eight pictures(Kim et al. 2010).

### Extracted curve features and age

**Fig. 6:**
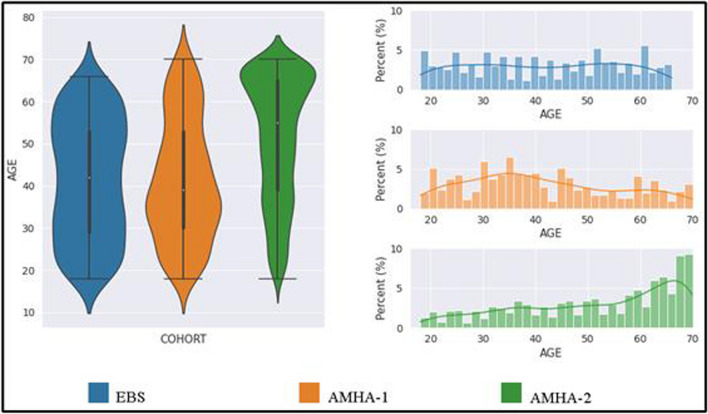
Violin plots for age. Violin plots for each of the three cohorts are provided for a visual comparison of the participant age distributions, along with the respective interquartile ranges (IQRs), and 95% CIs, with respect to the corresponding median across all cohorts

Distributions of age across the three samples showed generally flat distributions for the EBS and AMHA-1 cohorts, and a progressive increase in older subjects for AMHA-2 per violin plots (Fig. 6, Appendix). Univariate linear regressions between the 15 RPT features, and age were consequently run for the AMHA-2 cohort, showing significant effects, after correction for multiple comparisons. These results are listed in Table 8 (Appendix) for each regression, the standardized *β*, adjusted *R*^2^, and *p*-value associated with the overall regression was reported. Eleven out of the 15 RPT features showed trend effects with age. Only four features did not show a significant relationship with age after correction for multiple comparisons ($p<\frac{0.05}{15}\approx 0.0033$): *LA*, negative offset (*β*_*−*_), positive turning point (*ρ*_+_), and radial distance (*r*), which have implications for interpreting differences across the three cohorts, particularly where differences were suggested in RPT features between the EBS and AMHA-1 cohorts on one hand and the AMHA-2 cohort on the other. Overall, differences in features for the AMHA-2 cohort from the other two cohorts appear to be driven by differences in age between the cohorts.

## Discussion

Across the three distinct cohorts collected with the same pictures and procedures, this study found that: (1) picture ratings without an operant framework produced RPT curves with a similar mathematical form as those produced in an operant context (where each action has a consequence by changing the viewing time). (2) Rating-based RPT curves were discrete and recurrent across cohorts and scaled from individual to group data, meeting three of four criteria from Feynman (2017) for lawfulness. (3) RPT curves across the three cohorts showed high symmetry between liking and disliking assessment, which qualitatively differs from what is observed with operant keypress tasks. (4) Several features of these RPT curves were consistent across the three cohorts, but in some cases, differed relative to other experimental contexts, such as age. Of note, age still did not affect some RPT curve features such as *LA*, which is an observation that has previously been reported with keypress data (Lee et al. 2015).

As with the operant keypress procedure in other studies, the picture rating task produced data that showed RPT-like relationships (e.g., See Fig. 1). The rating task value functions, like those observed with keypressing, followed the pattern observed with prospect theory (Kahneman and Tversky 1979; Tversky and Kahneman 1992), and the limit functions followed those described by Markowitz (1952) for risk-reward curves. The value functions observed to date in RPT and prospect theory both calibrate “value” using a concave power function although the value function in RPT includes variables for both positive and negative preferences (e.g., K_+_ and K_−_), whereas the value function for prospect theory is based on one variable that could be either positive or negative. The quadratic function observed in prior RPT studies calibrates value intensity against its variance in RPT and Portfolio Theory (Kim et al. 2010; Lee et al. 2015; Livengood et al. 2017; Viswanathan et al. 2015, 2017). In both cases, this variance-mean function frames how value has a limit determined by variance measures. The same issue regarding having two preference measures for the variance-mean function in RPT and one for Portfolio theory is also relevant. In the current study using ratings, individual *R*^2^ values for both the value function and limit function graphs, across the three studies, ranged from 0.84 to 0.96 (Table 2), in line with previously published results in different cohorts using a keypress paradigm (Kim et al. 2010; Livengood et al. 2017; Viswanathan et al. 2017). Extracted features from the corresponding curves for the three rating experiments showed statistically consistent, and visually similar patterns. In particular, the 95% CI of the medians were broadly overlapping for the majority of RPT features, except for several differences between the EBS and AMHA-1 studies on one side and the AMHA-2 study on the other. Given the AMHA-2 study had a major difference in the proportion of older subjects (i.e., 55–70 years of age), and eleven of the 15 features used to compare cohorts were significantly associated with age for the AMHA-2 cohort, these differences likely relate to age distribution differences between cohorts.

The measure of *RA* did not statistically differ between cohorts, whereas the same metric applied to the avoidance curves (i.e., referred to as *LR* herein) statistically differed between the AMHA- 2 and the AMHA-1 and EBS cohorts, while the AMHA-1 and EBS cohorts were similar. Altogether, observations point to a greater symmetry in the valuing of positive (approach) and negative (avoidance) aspects of the stimuli when ratings are performed with no behavioral consequence, as opposed to prior studies based on keypressing. Supporting this observation, asymmetries in the mean–variance (*K,* *σ*) curves observed in prior operant keypress experiments (Livengood et al. 2017; Viswanathan et al. 2017) were not evident in individual or group data from the rating task. These observations with *RA* and *LR* raise a potential hypothesis for later study, namely that rating-based tasks may reflect a lower regard for negative consequences.

Although constructs such as Prospect Theory (Kahneman and Tversky 1979; Tversky and Kahneman 1992) consider the value function to be continuous with an inflection point, the rating task produced offsets, consistent with prior keypress experiments and RPT analyses (Kim et al. 2010; Livengood et al. 2017; Viswanathan et al. 2015, 2017). These offsets are suggestive of other psychological phenomena, such as the ante in poker, where a player must place a bet in the pot to enter the card game (e.g., *β*_+_), or an insurance premium paid to counter potential bad outcomes (e.g., *β*_*−*_). Further work is warranted to frame these findings.

Further research is also needed to deal with limitations to the current work, including that demographic matching between cohorts was not perfect, and the rating experiment used a unitary Likert-like scale as opposed to two scales wherein approach and avoidance assessments could be assessed independently. In line with caveat (1), we hypothesize demographic matching between cohorts may have contributed towards the statistical differences observed with the AMHA-2 when compared to AMHA-1 and EBS, such as differences in the distribution of age groups for AMHA-2 compared to the other cohorts. AMHA- 2 specifically contains a right-skewed distribution of participants from an older-age group demographic (Fig. 6, Appendix), whereas the distribution of age groups for AMHA-1 and EBS are more uniform. It should also be noted that our three population samples could still encode sample biases common to self-reported measures, such as response fatigue and framing effects. We sought to minimize these by keeping the overall task length short, and by allowing ratings to be bivalent with neutral instructions. This study did not use an operant design which is a core standard for connecting work to reward/aversion assessments and can be directly connected to the animal literature (Breiter et al. 1997). Despite this, it still produced interpretable and quantitative features of human judgment that were consistent, and have been shown in other studies to be quite important for the prediction of mental illness (e.g., Stefanopoulos et al. 2024; Lalvani et al. 2024; Bari et al. 2024). Lastly, the possibility of a Hawthorne effect (McCambridge et al. 2014) is likely limited by the anonymization of subjects with data collection, so subjects knew their responses could not be traced back to them.

With replication, the current findings, using cohorts calibrated to the U.S. Census, contributes to the big data psychology movement where the experimental environment cannot be as well-controlled as in a lab setting, but which can be conducted at a large-scale in much shorter time windows and with a major decrease in research team size and experimental time. Large-scale brain imaging and genetic studies are now quite common (e.g., The Connectome Project, ABCD Project, UK Brain Bank) and involve the collection of dense phenotyping data, although these studies are not primarily focused on human psychology and collect data over an extended time window with large human research teams. Studies with Amazon’s Mechanical Turk have argued for extension of task-based psychology studies to the web with small research teams (Buhrmester et al. 2016; Casler et al. 2013; Hauser and Schwarz 2016; Mason and Suri 2012; Paolacci et al. 2010), although there has been some critique of such practices (Chandler et al. 2019; Cheung et al. 2017; Crump et al. 2013). The current work points to the opportunity for testing computational behavior at larger scales than can be performed in the lab, allowing for greater sampling in the natural variance in measures.

In summary, the results of this study argue, that preference assessments in a psychological framework, made through a short and simple rating task, can be modelled quantitatively with *R*^2^ (goodness of fits) above 0.80, for RPT-based value functions, limit functions, and trade-off functions. Rating-based curves met three of the four strict criteria for lawfulness set out by Feynman (2017). These judgment features were consistent across three distinct experiments, suggesting a potential platform for large-scale study of population samples that can fuel machine learning applications, especially those in the domain of mental health (Bari et al. 2024; Stefanopoulos et al. 2024; Vike et al. 2024). Given the simplicity of the rating task application, its short duration and model-based analysis, this approach to preference quantitation might potentially be applied to the 84% of the world’s population that currently owns a smartphone (Turner 2022). This work demonstrates a simple framework for quantifying human preference at big data scale and allowing the interpretation of machine learning prediction through the use of cognitive science and neuroeconomic variables.

### Open practices statement

The data used for all experiments has been uploaded with this submission.

## Electronic supplementary material

Below is the link to the electronic supplementary material.

Supplementary file1 (XLSX 524 kb)

## Appendix

See Tables

**Table 5:** Demographics of rating experiment participants across three distinct cohorts observed in this study

Demographic	Group	Cohort Counts (Percentage %)		
		EBS	AMHA-1	AMHA-2
Gender identity	Male	225 (44.91)	262 (51.78)	1,592 (39.61)
	Female	276 (55.09)	243 (48.02)	2,408 (59.92)
	Other or prefer not to answer	0 (0.00)	1 (0.20)	19 (0.47)
Age group (years)	0–17	0 (0.00)	0 (0.00)	0 (0.00)
	18–24	79 (15.77)	67 (13.24)	245 (6.10)
	25–34	107 (21.36)	110 (21.74)	457 (11.37)
	35–44	90 (17.96)	128 (25.30)	603 (15.00)
	45–54	107 (21.36)	90 (17.79)	702 (17.47)
	55–64	102 (20.36)	73 (14.43)	977 (24.31)
	≥ 65	16 (3.19)	38 (7.51)	1,035 (25.75)
Education level	Some high school	19 (3.79)	12 (2.37)	112 (2.79)
	High school graduate	103 (20.56)	114 (22.53)	839 (20.88)
	Some college	201 (40.12)	114 (22.53)	1,172 (29.16)
	Bachelor’s degree	106 (21.16)	114 (22.53)	912 (22.69)
	Some graduate school	9 (1.80)	23 (4.55)	200 (4.98)
	Graduate degree	40 (7.98)	54 (10.67)	666 (16.57)
	Post-doctoral training	23 (4.59)	75 (14.82)	118 (2.94)
Handedness	Right	423 (84.43)	393 (77.67)	3,446 (85.74)
	Left	70 (13.97)	80 (15.81)	478 (11.89)
	Both	8 (1.60)	33 (6.52)	95 (2.36)
Race ethnicity	White/Caucasian	353 (70.46)	355 (70.16)	3,340 (83.11)
	African American	80 (15.97)	61 (12.06)	277 (6.89)
	Hispanic/Latinx	20 (3.99)	34 (6.72)	139 (3.46)
	Asian or Pacific Islander	16 (3.19)	15 (2.96)	145 (3.61)
	Native American or Alaskan Native	6 (1.20)	7 (1.38)	28 (0.70)
	Mixed racial background	24 (4.79)	27 (5.34)	37 (0.92)
	Other race	1 (0.20)	4 (0.79)	21 (0.52)
	Prefer not to answer	1 (0.20)	3 (0.59)	32 (0.80)

Demographic data for subject populations studied by rating task is shown per cohort. For each demographic, group counts and their relative percentages within the respective cohort are provided

5,

**Table 6:** Mathematical terms with their respective RPT term and symbol

Mathematical term	RPT term	Symbol
Loss Aversion	Loss Aversion	LA
Risk Aversion	Risk Aversion	RA
Loss Resilience	Loss Resilience	LR
Positive Offset	Ante	β_+_
Negative Offset	Insurance	β_–_
Positive Apex	Peak Positive Risk	α_−_
Negative Apex	Peak Negative Risk	α_−_
Positive Turning Point	Reward Tipping Point	ρ_+_
Negative Turning Point	Aversion Tipping Point	ρ_-_
Positive Quadratic Area	Total Reward Risk	q_+_
Negative Quadratic Area	Total Aversion Risk	q_−_
Mean Polar Angle	Reward-Aversion Tradeoff	θ
Polar STD	Tradeoff Range	σ_θ_
Mean Radial Distance	Reward-Aversion Consistency	r
Radial STD	Consistency Range	σ_r_

6,

**Table 7:** Picture ratings summary statistics across cohorts

Category	Sum of ratings (Mean rating per picture)					
	Avoidance (Negative ratings)			Approach (Positive ratings)		
	EBS	AMHA-1	AMHA-2	EBS	AMHA-1	AMHA-2
Aggressive animals	− 6748 (− 2.3354)	− 4784 (− 2.3719)	− 65,383 (− 2.5629)	1102 (1.7225)	3024 (1.9194)	6686 (1.9861)
Nature	− 352 (− 1.7325)	− 466 (− 1.6944)	− 3013 (− 1.8483)	7460 (2.2487)	7527 (2.2516)	60,939 (2.3263)
Cute animals	− 396 (− 1.7071)	− 456 (− 1.7757)	− 3723 (− 1.8818)	8058 (2.3523)	8111 (2.3284)	66,348 (2.4463)
Disaster	− 7203 (− 2.4161)	− 5255 (− 2.4392)	− 68,694 (− 2.5960)	940 (1.7608)	2904 (1.9926)	5117 (1.8846)
People in bathing suits	− 3092 (− 2.1251)	− 1697 (− 2.1025)	− 23,676 (− 2.2657)	3529 (2.1478)	5227 (2.0948)	26,998 (2.1937)
Sports	− 1700 (− 1.8305)	− 1298 (− 1.8180)	− 13,971 (− 1.9396)	3657 (1.7883)	5248 (1.9844)	28,672 (1.9020)

Summary and descriptive statistics for the “total sum responses across the cohort” and “mean response”, stratified by positive or negative valence of ratings (approach, +, or avoidance, −). The total sum of approach (+) and avoidance (−) responses is shown per cohort for each category of picture shown from the IAPS stimulus set, along with the average response for each picture, per category

7,

**Table 8:** Summary statistics of univariate linear regression across each RPT feature versus age for AMHA-2 cohort

RPT metric	Standardized *β*	*R*2	*p*-value
Loss aversion (LA)	5.331 × 10^–3^	−2.614 × 10^−4^	0.7542
Risk aversion (RA)	0.1046	1.064 × 10^−2^	1.373 × 10^−9^
Loss resilience (LR)	0.1212	1.439 × 10^−2^	2.675 × 10^−12^
Positive offset (*β*_+_)*	−9.869 × 10^−2^	9.441 × 10^−3^	1.266 × 10^−8^
Negative offset (*β*_*-*_)*	2.272 × 10^−2^	2.210 × 10^−4^	0.1862
Positive apex (*α*_+_)*	− 0.1098	1.175 × 10^−2^	1.898 × 10^−10^
Negative apex (*α*_+_)*	5.176 × 10^−2^	2.378 × 10^−3^	2.859 × 10^−3^
Positive turning point (*ρ*_+_)*	5.328 × 10^−3^	−2.667 × 10^−4^	0.7564
Negative turning point (*ρ*_*-*_)*	9.564 × 10^−2^	8.857 × 10^−3^	2.193 × 10^−8^
Positive quadratic area (*q*_+_)*	−9.996 × 10^−2^	9.629 × 10^−3^	5.095 × 10^−9^
Negative quadratic area (*q*_*-*_)*	6.124 × 10^−2^	3.462 × 10^−3^	3.117 × 10^−4^
Polar angle (*θ*)	−8.792 × 10^−2^	7.429 × 10^−3^	4.259 × 10^−7^
Polar dispersion (*σ*_*θ*_)	0.2123	4.479 × 10^−2^	1.789 × 10^−34^
Radial distance (*r*)	−4.306 × 10^−2^	1.554 × 10^−3^	1.302 × 10^−2^
Radial dispersion (*σ*_*r*_)	0.1744	3.012 × 10^−2^	4.255 × 10^−24^

Unstarred features of the (K, H), (K, σ), and (H−, H+) curves are in dimensionless units, facilitating comparison across cohorts

8, Figs. ^5^, 6.
